# Mechanism for Green Development Behavior and Performance of Industrial Enterprises (GDBP-IE) Using Partial Least Squares Structural Equation Modeling (PLS-SEM)

**DOI:** 10.3390/ijerph17228450

**Published:** 2020-11-15

**Authors:** Xingwei Li, Jianguo Du, Hongyu Long

**Affiliations:** 1 School of Management, Jiangsu University, Zhenjiang 212013, China; 2111710001@stmail.ujs.edu.cn; 2 College of Architecture and Urban-Rural Planning, Sichuan Agricultural University, Chengdu 611830, China; 3 School of Civil Engineering and Geomatics, Southwest Petroleum University, Chengdu 610500, China; 201821000820@stu.swpu.edu.cn

**Keywords:** industrial ecology, green development, organizational behavior, environmental economics, green supply chain management practice, partial least squares structural equation modeling (PLS-SEM)

## Abstract

Although the theory of green development behavior and performance of industrial enterprises (GDBP-IE) reveals that the green development behavior (GDB) of industrial enterprises is affected by internal and external factors and produces performance, it lacks a clear mechanism. This paper aims to verify the theory of GDBP-IE and reveals the mechanism of GDBP-IE in the Chinese context. The partial least squares structural equation modeling (PLS-SEM) method was used to analyze valid samples of Chinese industrial enterprises (*N* = 615). The empirical conclusions are as follows. (1) Corporate tangible resources, corporate intangible resources (CIR), market environment, public supervision and policy and institutional environment (PIE) have a significant positive effect on GDB (i.e., green supply chain management practice and clean production behavior). (2) Compared with other factors, the positive effect of CIR on GDB is the strongest. (3) The level of positive effect of PIE on GDB is not as significant as other factors. (4) GDB has a significant positive effect on green development performance (i.e., corporate social performance, corporate financial performance and corporate environmental performance). This paper provides effective evidence for researchers to use other methods to further verify the theory of GDBP-IE in the Chinese context. This paper also provides an opportunity for cluster analysis of GDBP-IE in different countries or regions. In addition, this paper not only gives a targeted reference for the government to formulate guidelines concerning the green development of industrial enterprises but also encourages industrial enterprise managers to formulate green development strategies, which is a way to help industrial enterprise managers and workers to participate in and comply with GDB.

## 1. Introduction

Green development is a complex adaptive system composed of legal policy systems, production systems and living systems and is closely related to society, economy and the natural environment. The community of human and natural life is at the core of green development [1,2]. The green development of industrial enterprises, a major concern of industrial ecology and environmental economics researchers, regards industrial enterprises as the main body, e.g., the effect of the market mechanism on the green development of industrial enterprises [3], the effect of industrial agglomeration on green development efficiency [4], the influencing mechanism of technology R&D on green development in China’s industrial sector [5] and the evaluation framework of industrial green development [6,7]. The production activities of industrial enterprises belong to the production subsystem of the green development system, which are not only affected by the policy subsystem, but also provide necessary products and services for the living subsystem. However, industrial pollution is an inevitable product in the production activities of industrial enterprises. Fortunately, the Chinese government attaches great importance to the green development of industrial enterprises and has adopted a series of measures. According to data from the ministry of ecology and environment of the People’s Republic of China [8], among different types of direct sea pollution sources, the sewage discharge volume of industrial pollution sources ranks second only to comprehensive sewage outlets. Ninety-five percent of the industrial parks at or above the provincial level in the Yangtze River Economic Zone have built sewage treatment facilities and installed online monitoring devices. China’s total investment in the treatment of industrial pollution sources has remained stable and averaged RMB 81.79 billion (approximately US$11.688 billion) from 2014 to 2017 [9]. Nevertheless, the current industrial pollution is still difficult to eliminate. Since 2019, coronavirus disease 2019 (COVID-19), which has more cases and deaths than severe acute respiratory syndrome (SARS), has spread into countries and regions around the world [10,11]. Due to the spread of COVID-19, industrial enterprises in countries and regions around the world have been negatively affected in varying degrees, e.g., economic recession [12], disrupting the supply chain [13,14], stopping production [15], etc. In the context of industrial pollution and the COVID-19 epidemic, how can the production activities of industrial enterprises be resumed as soon as possible? Moreover, how can the production activities of industrial enterprises be made to be more in line with the framework of green development? An adequate assessment of the mechanism for green development behavior (GDB) and performance of industrial enterprises (GDBP-IE) is required to give a scientific judgment.

This paper aims to make up for the lack of empirical research on the theory of GDBP-IE in existing research and to reveal the mechanism of GDBP-IE in the Chinese context. Its scientific questions are how do influencing factors affect the GDB of industrial enterprises and how does the GDB of industrial enterprises affect the green development performance of industrial enterprises?

To the best of our knowledge, this paper uses the partial least squares structural equation modeling (PLS-SEM) method for the first time to verify the theory of GDBP-IE in the Chinese context and reveal its mechanism. This paper has both theoretical and practical significance. (1) Its theoretical significance is, first, that it enriches the literature in the fields of green development, industrial ecology, environmental economics and organizational behavior. This is conducive to the integration of green development, industrial ecology, environmental economics and organizational behavior, breaking through the limitations of previous single research. Second, this paper provides effective evidence for researchers to use other methods to further verify the theory of GDBP-IE in the Chinese context. This paper not only considers the generality of the theory of GDBP-IE but also combines the particularity of industrial enterprises in the Chinese context. Third, this paper contributes a theoretical basis for researchers to verify the theory of GDBP-IE in the context of other countries or regions, thus providing an opportunity for cluster analysis of GDBP-IE in different countries or regions. Fourth, researchers of agricultural and service industries can refer to this paper to explore and verify the mechanism of green development behavior performance. (2) This paper’s practical significance is, first, that it gives a targeted reference for the government to formulate guidelines for the green development of industrial enterprises. Second, this paper provides a valuable basis for industrial enterprise managers to formulate green development strategies. Third, it is a way to help industrial enterprise managers and workers to participate in and comply with GDB.

The remaining sections are organized as follows: Section 2 includes a literature review, hypotheses development and the GDBP-IE hypothetical model. The PLS-SEM approach and data collection and sampling are introduced in Section 3. The results of tests of the global and measurement models are analyzed in Section 4. Discussion and conclusions are provided in Section 5 and Section 6, respectively.

## 2. Literature Review and Hypotheses Development

The theory of GDBP-IE was first proposed by Li et al. [16]. The theory states that in the process of development, industrial enterprises form two specific organizational behaviors in response to environmental protection, i.e., clean production behavior (CPB) and green supply chain management practice (GSCMP). These two behaviors are called the GDB of industrial enterprises and belong to the scope of green behavior [17]. Cleaner production is a preventive environmental protection measure for companies to apply the concept of environmental sustainability to manufacture more products with less cost and to reduce risks to humans and the environment [18,19]. Cleaner production has received extensive attention from researchers in many industrial enterprises on various topics, e.g., cleaner production in mines [20] and cleaner production in the steel industry [21]. CPB refers to organizational behavior formed by the enterprise in the process of cleaner production [16,22]. The supply chain includes all links (i.e., customers, retailers, wholesalers or distributors, manufacturers, suppliers of parts or raw materials) directly or indirectly involved in the process of meeting customer needs. Each link of the supply chain is interconnected through product flow, information flow and capital flow. In order to satisfy the ultimate customers of the supply chain, supply chain management must unify procurement, manufacturing, marketing, logistics and information systems [23,24,25] and coordinate the strategy of customer-centricity, efficiency, quality and responsiveness [26]. As the concept of green development has been widely accepted, industrial companies have responded to customers’ legitimate demands for greening products and services by implementing GSCMP [27,28]. Existing research clearly defines the concept, research object and research scope of the GDB of industrial enterprises. In particular, Li et al. [22] developed a 70-item scale based on the theory of GDBP-IE, providing a tool for quantitative research on GDBP-IE. Unfortunately, there is limited research verifying the mechanism among the variables in GDBP-IE. Specifically, how do corporate tangible resources (CTR), corporate intangible resources (CIR), market environment (ME), public supervision (PS), policy and institutional environment (PIE) affect GDB? How does GDB affect green development performance? Are the degrees of these effects consistent?

### 2.1. Green Development Behavior (GDB) and Factors

The theory of GDBP-IE points out that the internal factors of GDB of industrial enterprises include two parts, i.e., CTR [29,30] and CIR [31,32]. The external factors of GDB of industrial enterprises include three parts, i.e., ME [33,34,35], PS [36,37] and PIE [38]. The theory of GDBP-IE is supported by other theories. First, consistent with the viewpoints of the resource-based view of the firm [30] and resource-based theory of the firm [31], the theory of GDBP-IE believes that enterprise resources include CTR and CIR. A resource-based view of the firm and resource-based theory are two different theories. The resource-based view of the firm was a theory proposed by Wernerfelt in 1984 [39]. Within the conceptual framework of the resource-based view of the firm, resources are considered to be anything that brings advantages or disadvantages to the enterprise, including tangible and intangible resources. From this perspective, resources are used to help firms make strategic decisions, and from the perspective of resources, the resources that bring firms high profits can be identified [40]. Resource-based theory was proposed by Conner in 1991 [41]. Under the conceptual framework of resource-based theory, although enterprises are seekers of costly-to-copy inputs for production and distribution, they are subject to internal and external constraints. Moreover, the difference between enterprises depends on the investment of resources. Although the resource-based view of the firm and resource-based theory are different, they strongly support the internal factors in the theory of GDBP-IE. Second, the theory of GDBP-IE and the theory of industrial organization [33,34,35] agree that ME, PS and PIE affect industrial enterprises. In the conceptual framework of the theory of industrial organization, the paradigm called “structure-conduct-performance” was developed according market structure (the number of sellers in the market, their degree of product differentiation, the cost structure, the degree of vertical integration with suppliers, etc.). The start of industrial organization is the structure and behavior of the enterprise (market strategy and internal organization), but it is also subject to supervision by outsiders (including the government and the public) [42]. Therefore, this theory strongly supports the external factors in the theory of GDBP-IE. In order to verify the mechanism of CTR, CIR, ME, PS and PIE on GDB, we propose the following five hypotheses:

**Hypothesis 1.** *CTR has a significant positive effect on GDB.*

**Hypothesis 2.** *CIR has a significant positive effect on GDB.*

**Hypothesis 3.** *ME has a significant positive effect on GDB.*

**Hypothesis 4.** *PS has a significant positive effect on GDB.*

**Hypothesis 5.** *PIE has a significant positive effect on GDB.*

### 2.2. GDB and Green Development Performance

In addition, the theory of GDBP-IE points out that the green development performance of industrial enterprises includes three parts, i.e., corporate social performance (CSP), corporate financial performance (CFP) and corporate environmental performance (CEP) [43,44,45,46]. In order to measure corporate sustainability performance, Elkington proposed a framework in 1997, which is called the triple bottom line [47]. Since the triple bottom line was proposed, it has been recognized by many researchers concerned with enterprises. Especially in the division of corporate performance, the triple bottom line supports performance in the theory of GDBP-IE. Thus, the theories of GDBP-IE and triple bottom line [43,44] are consistent in that they both explain performance from three aspects, i.e., financial, environmental and social performance. In order to verify the mechanism of GDB on green development performance, we propose the following hypothesis:

**Hypothesis 6.** *GDB has a significant positive effect on green development performance.*

The theory of GDBP-IE has received widespread attention since its emergence. It is applied to support various research viewpoints, e.g., the social performance of mining firms [48], green investment decisions of the manufacturer [49], green development system models [1], green development reporting framework for enterprises [50], identification of critical factors in construction and demolition waste recycling [51] and renewable energy and green economic growth [52]. Researchers have used the theory of GDBP-IE in many areas of industrial enterprises, but these studies cannot clearly reveal the mechanism of GDBP-IE. Therefore, this paper can make up for this shortcoming.

**Figure 1:**
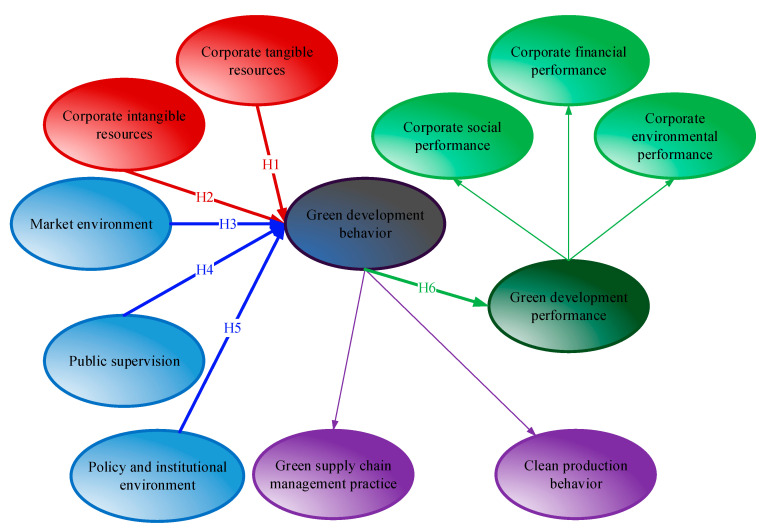
The green development behavior and performance of industrial enterprises (GDBP-IE) hypothetical model.

Figure 1 shows the GDBP-IE hypothetical model. From the above six hypotheses, GDB comprises two behaviors, namely CPB and GSCMP, which are two lower-order constructs (LOCs) that make up a higher-order construct (HOC). The same occurs with green development performance, which in turn is made up of three LOCs as subcomponents: CSP, CFP and CEP. According to Hair et al. [53], this model is designed as a hierarchical component model (HCM) and is assessed and analyzed as such.

## 3. Research Methods

### 3.1. Squares Structural Equation Modeling (PLS-SEM)

Structural equation modeling is a multivariate analysis method used to assess the consistency of hypothetical models and collected samples with a theory [54,55]. Covariance-based structural equation modeling (CB-SEM) and partial least squares structural equation modeling (PLS-SEM) are the main techniques of structural equation modeling [56,57,58]. Compared with CB-SEM, PLS-SEM is more flexible in specifying the relationship between items and constructs for researchers [59]. PLS-SEM works well with any sample size as long as it meets the minimum sample size requirements, and it allows for putting forward hypotheses for the variables that have complex effects on specific aspects of the model. PLS-SEM works with composites (latent variables or constructs) whose measurement models can be operationalized as mode A (previously called reflective), mode B (previously called formative) or the common factor. Therefore, the PLS-SEM approach is widely used by researchers [60].

The advantages of the PLS-SEM approach are as follows. First, it can be verified using PLS-SEM [61]. Second, according to a few observations, PLS-SEM can be used to reliably estimate complex hypothetical models [62]. PLS-SEM works well in any scenario, especially with very complex models made up of many latent variables and many indicators. However, its aim is to obtain models that are as parsimonious as possible [62]. At present, PLS-SEM has been successfully applied in many fields of social science research, e.g., hospitality management [63], construction industry [64], competitive performance [65], organization and the environment [66]. Therefore, the PLS-SEM approach was used to verify our six proposed hypotheses.

The specific steps are as follows. First, Smartpls v3.2.1 (SmartPLS GmbH, Bönningstedt, Germany) [67] software was used to verify the fitting index of the measured model. Second, to test common method bias, SPSS v25 (IBM SPSS Inc., Chicago, IL, USA) software was used to perform a Harman’s single factor test. Third, to test multicollinearity, variance inflation factor (VIF) was used to evaluate the multicollinearity problems. Fourth, to test reliability, four indicators were tested, i.e., standardized indicator loadings (SIL), cronbachs alpha (CA), composite reliability (CR) and average variance extracted (AVE). In addition, R^2^ was used to test the degree of explained variance of the endogenous variables. Fifth, the heterotrait-monotrait ratio (HTMT) was used to complete the discriminant validity test [68]. Finally, path coefficients and confidence intervals were reported.

### 3.2. Data Collection and Sampling

This paper used a GDBP-IE scale with 70 items as the questionnaire [22]. Table 1 shows the structure of the GDBP-IE scale. In Appendix A, Table A1, Table A2, Table A3 and Table A4 gives all items of the GDBP-IE scale.

Using a random sampling strategy, 700 questionnaires were issued to industrial workers and managers from 31 provinces in China (excluding Hong Kong, Macau and Taiwan) through online surveys using a commissioned questionnaire website www.wjx.cn. The survey time was March 2020. After excluding invalid questionnaires, 615 valid questionnaires were obtained (effective rate reached 87.9%). The measured model has 10 latent variables and 70 observable variables. It satisfies the criterion for the sample size in SEM of being at least five times the number of the measurable item [69]. In addition, according to Clemente et al. [70] and Maccallum and Bryant [71], the minimum sample size was estimated using Gpower software (University of Dusseldorf, Dusseldorf, Germany). The size of the sample was calculated to ensure α of 0.05 and power at 1-β = 0.80, and the result indicated a sample of 279, which is less than we have included (*N* = 615). Table 2 gives the descriptive statistics of the sample.

According to Table 2, the frequencies of males and females in the sample were 365 (59.35%) and 250 (40.65%). Therefore, in the sample gender structure, the proportion of males was slightly higher than that of females, which is in line with the characteristics of Chinese industrial enterprises. The frequencies in age of <30, 30–39, 40–49 and >50 in the sample were 190 (30.89%), 257 (41.79%), 128 (20.81%) and 40 (6.5%), respectively. Therefore, the age structure of the sample was mainly middle-aged and young, which is in line with the characteristics of Chinese industrial firms. The frequencies in the positions of worker and manager in the sample were 252 (40.98%) and 363 (59.02%), respectively. Therefore, in the position structure of the sample, the proportion of managers was slightly higher than that of workers, which represents the GDB of industrial enterprises in terms of organizational behavior attributes. The frequencies in the level of education of bachelor’s degree and the others in the sample were 379 (61.63%) and 236 (38.37%), respectively, which are in line with the Chinese context. In the number of employees in the enterprise, the frequencies of <300, 301–1000 and >1000 were 203 (33.01%), 260 (42.28%) and 152 (24.72%), respectively. Therefore, the scale of industrial enterprises in the sample covers large, medium and small companies. In summary, the sample is representative.

## 4. Result

### 4.1. Tests of Global Model Fit

According to the recommendations of Bagozzi and Yi [72], root mean square error of approximation (RMSEA) was used to test the fit indices for the global model. Generally, when the value of RMSEA is less than 0.05, it represents good fit. When the value is between 0.05 and 0.08, it has fair model fit [73]. The results show that the value of RMSEA was 0.047, which meets the requirement for the value of RMSEA indicating good fit. In order to avoid the common method bias problem, SPSS v25 (IBM SPSS Inc., Chicago, IL, USA) software was used to perform a Harman’s single factor test. The first factor accounted for 40.007% of the variation, a value below 50% is the threshold of the common method bias [74,75]. Existing research proposed that if the VIF does not exceed 3.3, then there are no multicollinearity problems [61]. The values of VIF were between 1.0 and 1.547. Therefore, the fit indices for the global model are acceptable, and there is no common method bias or multicollinearity problems.

#### 4.1.1. Reliability Analysis

The measured model was evaluated through reliability and validity tests. In the reliability test, four indicators were tested, i.e., SIL, CA, CR and AVE.

(1) The values of SIL [76], CA [77] and CR [72] all must be above 0.70. (2) The AVE of every construct should be above 0.5 [78,79,80]. The coefficient of determination (R^2^) represents the degree of explained variance of the endogenous variables [81]. The R^2^ is used to determine the explanatory power of a structural model [82]. The R^2^ must be satisfactory, where values of 0.25, 0.50 and 0.75 for target constructs are considered weak, medium and substantial, respectively [80]. Table 3 shows the test results of reliability and R^2^.

Table 3 shows that the SIL value was between 0.700 and 0.949 (above 0.70), the CA value was between 0.859 and 0.948 (above 0.70), the CR value was between 0.928 and 0.990 (above 0.70), the AVE value was between 0.681 and 0.898 (above 0.50) and the R^2^ value was between 0.499 and 0.845 (above 0.25).

#### 4.1.2. Discriminant Validity Analysis

The heterotrait-monotrait ratio (HTMT) [69] of correlations was used to complete the discriminant validity test (Table 4). Compared with the Fornell–Larcker criterion and (partial) cross-loadings, the heterotrait–monotrait ratio of correlations is superior. The recommended threshold (0.9) was used as the criterion.

The findings of the measured model showed that the model had good reliability, convergence validity and discriminant validity and confirmed that the constructs were statistically diverse.

### 4.2. Tests of the Measurement Models

**Figure 2:**
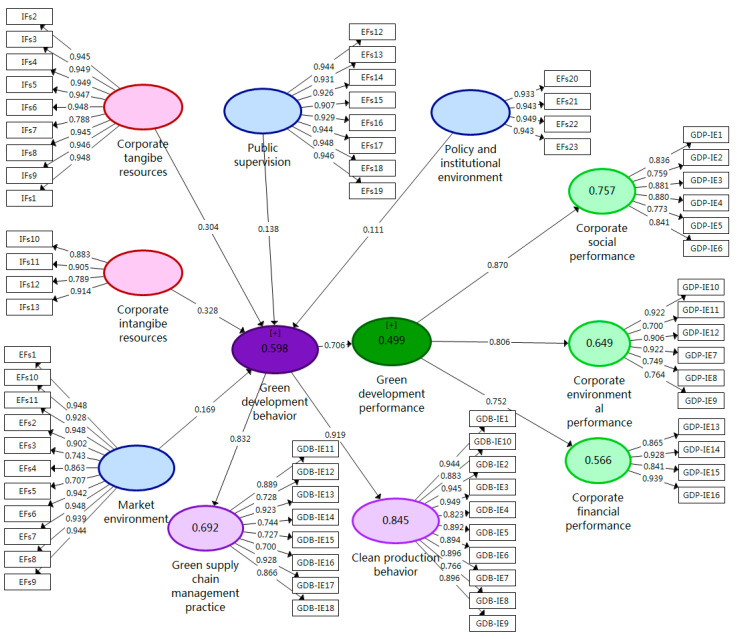
Tests of the measurement models.

Measurement models include common factors, that is, (1) GDB includes GSCMP and CPB and (2) GDP includes CSP, CEP and CFP. Figure 2 shows the results of tests of the measurement models after applying the consistent PLS algorithm. For these higher-order constructs (HOCs), a repeated indicators approach has been used to estimate the latent variables scores. Effect size is used to measure whether an independent variable has a substantial influence on a dependent variable [83]. An effect size (*f*^2^) of 0.02 is regarded as small, 0.15 is moderate and 0.35 is strong [84]. Table 5 shows that the effect sizes between variables were moderate and strong.

First, as with the initial model estimation, we retained all missing value processing and PLS-SEM algorithm settings. Next, the “No Sign Changes” and “Complete Bootstrapping” option was selected to run the 5000 bootstrap samples. Finally, bias correction and acceleration (BCa) was used in the bootstrapping procedure, and a one-tailed test with a significance level of 0.05 was performed in the advanced settings. Table 5 shows that all of the hypotheses are supported (i.e., H1, H2, H3, H4, H5 and H6), among which the effect for two supported hypotheses (i.e., H2 and H6) is strong and the effect for four supported hypotheses (i.e., H1, H3, H4 and H5) is moderate.

## 5. Discussion

To the best of our knowledge, this paper uses the PLS-SEM approach for the first time to verify the theory of GDBP-IE in the Chinese context and reveal the mechanism. Although this empirical study is based on the Chinese context, the findings have effectively verified the theory of GDBP-IE. In particular, in the context of industrial pollution and the COVID-19 epidemic, this research helps industrial companies improve their performance through GDB.

(1) This paper found that the internal factors (i.e., CTR and CIR) and external factors (i.e., ME, PS and PIE) have significant positive effects on GDB. Wang et al. [85] found that the cost of CTR and the customer of ME are positively correlated with GSCMP. Jabbour et al. [86] indicated that the management system of CIR (such as environmental management maturity) is positively correlated with GSCMP. Liu et al. [87] suggested that the external pressures from regulations is positively correlated with GSCMP. Laosirihongthong et al. [88] reported that the threat of legislation and regulation can improve GSCMP. These research results support our findings. Some researchers divided the company’s resources into CTR and CIR just like our research [89], and some research, like ours, showed that ME can have an impact on CPB [90,91]. Nonetheless, they did not clearly point out the mechanism of CTR and CIR on GDB. This empirical study effectively made up for this regret.
(2) This paper found that GDB has a significant positive effect on green development performance (i.e., CSP, CEP and CFP). Azevedo et al. [92] reported that GSCMP can improve CSP. Green et al. [93] found that GSCMP can improve CEP and CFP. These research results support our findings. Although some research findings revealed that CPB can form CSP, CEP and CFP, there is no clear indication of the mechanism [1,16,94]. This empirical study effectively made up for this regret.
(3) This paper found that the level of positive effect of PIE on GDB is not as significant as other factors. This result may be related to the motivation of GDB. According to the theory of corporate social responsibility, companies will take into account the expectations of stakeholders and triple performance in a specific context and then take a series of actions [95]. For enterprises, corporate social responsibility is also an opportunity for development. Compared to PS and PIE, customers in ME generally prefer products formed through GDB. As far as the degree of sales in the ME is concerned, products formed through GDB and non-GDB are completely different. Therefore, GDB may be a means of competition between enterprises. In other words, companies have the motivation to actively participate in and comply with GDB. This may be the reason why the level of the positive effect of PIE on GDB is not as significant as other factors.

## 6. Conclusions

According to the theory of GDBP-IE, this paper reveals the mechanism of GDBP-IE in the Chinese context. The PLS-SEM approach was used to analyze valid samples of Chinese industrial enterprises (*N* = 615). The empirical conclusions are as follows.

(1) CTR, CIR, ME, PS and PIE have a significant positive effect on GDB (i.e., CPB and GSCMP).
(2) Compared with other factors, the positive effect of CIR on GDB is the strongest.
(3) The level of positive effect of PIE on GDB is not as significant as other factors.
(4) GDB has a significant positive effect on green development performance (i.e., corporate social performance, corporate financial performance and corporate environmental performance).

Based on the above conclusions, the following three implications can be derived.

(1) Since CTR, CIR, ME, PS and PIE have significant positive effects on GDB, actions can be taken accordingly. Under the premise of considering GDB (i.e., CPB and GSCMP), industrial enterprises should not only increase their investment in tangible and intangible resources (e.g., fixed assets, human resources, technical resources, culture and management systems), but also pay attention to the influence of stakeholders and actively respond to government regulations, public supervision and demand and the capabilities of competitors.
(2) Compared with other factors, the positive effect of CIR on GDB is the strongest. Therefore, under the premise of considering GDB, enterprises should give priority to increasing investment in intangible resources (e.g., technical resources, culture and management systems).
(3) GDB has significant positive effects on green development performance (i.e., CSP, CEP and CFP). Therefore, under the premise of considering green development performance (i.e., CSP, CEP and CFP), industrial enterprises should actively participate in and comply with GDB (i.e., GSCMP and CPB). Because GDB has a particularly prominent positive effect on green development performance, industrial enterprises should give priority to satisfying GDB to improve their own green development performance.
(4) Currently, countries around the world are subject to industrial pollution and the COVID-19 epidemic. Industrial enterprises should pay full attention to CTR, CIR, ME, PS and PIE. In addition, industrial companies should actively participate in and comply with GDB in order to achieve GDP.

Like most studies, there are some limitations to this paper. Composite reliability values above 0.90 (and definitely above 0.95) are not desirable because they indicate that all manifest variables (items) are measuring the same phenomenon and are therefore not likely to be a valid measure of the construct. Specifically, such composite reliability values occur if one uses semantically redundant items by slightly rephrasing the very same question. This is also because SILs are so high. It is recommended to take this into account in further research. Although this paper effectively verified the theory of GDBP-IE and the mechanism in the Chinese context, other countries and regions were not considered. Is the mechanism of GDBP-IE in other countries and regions homogeneous or heterogeneous with the Chinese context? In particular, what are the similarities and differences in the mechanism of GDBP-IE between developed and developing countries? In addition, the conclusions of this paper only considered industrial enterprises, and enterprises in the agricultural and service industries were not considered. Compared with GDBP-IE, is the GDB performance of agricultural enterprises and service industry enterprises homogeneous or heterogeneous in terms of mechanism? These issues are not only the limitations of this paper but also opportunities for other researchers to study further. We suggest that researchers further explore and verify these issues on the basis of our conclusions.

## Figures and Tables

**Table 1:** Structure of the GDBP-IE scale.

Sub-Scale	Dimensions	No. of Items
Internal Factors (IFs)		13
	Corporate tangible resources (CTR)	9
	Corporate intangible resources (CIR)	4
External Factors (EFs)		23
	Market environment (ME)	11
	Public supervision (PS)	8
	Policy and institutional environment (PIE)	4
Green Development Behavior of Industrial Enterprises (GDB-IE)		18
	Clean production behavior (CPB)	10
	Green supply chain management practices (GSCMP)	8
Green Development Performance of Industrial Enterprises (GDP-IE)		16
	Corporate social performance (CSP)	6
	Corporate financial performance (CFP)	6
	Corporate environmental performance (CEP)	4

**Table 2:** Sample demographics (*N* = 615).

Socio-Demographic Factors		Frequency	Proportion
**Gender**			
	Male	365	59.35%
	Female	250	40.65%
**Age**			
	<30	190	30.89%
	30–39	257	41.79%
	40–49	128	20.81%
	>50	40	6.50%
**Position**			
	Worker	252	40.98%
	Manager	363	59.02%
**Level of education**			
	Bachelor’s degree	379	61.63%
	Other	236	38.37%
**Number of employees in the enterprise**			
	<300	203	33.01%
	301–1000	260	42.28%
	>1000	152	24.72%

**Table 3:** Reliability, convergent validity and R^2^.

Constructs		Path Relationships	SIL	CA	CR	AVE	R^2^	
							Value	LEP
CTR				0.945	0.994	0.898	-	-
		IFs1 ← CTR	0.948					
		IFs2 ← CTR	0.945					
		IFs3 ← CTR	0.949					
		IFs4 ← CTR	0.949					
		IFs5 ← CTR	0.947					
		IFs6 ← CTR	0.945					
		IFs7 ← CTR	0.788					
		IFs8 ← CTR	0.945					
		IFs9 ← CTR	0.946					
CIR				0.859	0.928	0.764	-	-
		IFs10 ← CIR	0.883					
		IFs11 ← CIR	0.905					
		IFs12 ← CIR	0.789					
		IFs13 ← CIR	0.914					
ME				0.946	0.99	0.752	-	-
		EFs1 ← ME	0.948					
		EFs2 ← ME	0.902					
		EFs3 ← ME	0.743					
		EFs4 ← ME	0.863					
		EFs5 ← ME	0.707					
		EFs6 ← ME	0.942					
		EFs7 ← ME	0.948					
		EFs8 ← ME	0.939					
		EFs9 ← ME	0.944					
		EFs10 ← ME	0.928					
		EFs11 ← ME	0.948					
PS				0.941	0.99	0.86	-	-
		EFs12 ← PS	0.944					
		EFs13 ← PS	0.931					
		EFs14 ← PS	0.926					
		EFs15 ← PS	0.907					
		EFs16 ← PS	0.929					
		EFs17 ← PS	0.944					
		EFs18 ← PS	0.948					
		EFs19 ← PS	0.946					
PIE				0.948	0.97	0.887	-	-
		EFs20 ← PIE	0.933					
		EFs21 ← PIE	0.943					
		EFs22 ← PIE	0.949					
		EFs23 ← PIE	0.943					
GDB-IE				0.946	0.997	0.841	0.598	Medium
	CPB			0.942	0.992	0.841	0.845	Substantial
		GDB-IE1 ← CPB	0.944					
		GDB-IE2 ← CPB	0.945					
		GDB-IE3 ← CPB	0.949					
		GDB-IE4 ← CPB	0.823					
		GDB-IE5 ← CPB	0.892					
		GDB-IE6 ← CPB	0.894					
		GDB-IE7 ← CPB	0.896					
		GDB-IE8 ← CPB	0.766					
		GDB-IE9 ← CPB	0.896					
		GDB-IE10 ← CPB	0.883					
	GSCMP			0.927	0.971	0.681	0.692	Medium
		GDB-IE11 ← GSCMP	0.889					
		GDB-IE12 ← GSCMP	0.728					
		GDB-IE13 ← GSCMP	0.923					
		GDB-IE14 ← GSCMP	0.744					
		GDB-IE15 ← GSCMP	0.727					
		GDB-IE16 ← GSCMP	0.700					
		GDB-IE17 ← GSCMP	0.928					
		GDB-IE18 ← GSCMP	0.866					
GDP-IE				0.926	0.994	0.706	0.499	Medium
	CSP			0.909	0.955	0.706	0.757	Substantial
		GDP-IE1 ← CSP	0.836					
		GDP-IE2 ← CSP	0.759					
		GDP-IE3 ← CSP	0.881					
		GDP-IE4 ← CSP	0.880					
		GDP-IE5 ← CSP	0.773					
		GDP-IE6 ← CSP	0.841					
	CEP			0.908	0.955	0.711	0.649	Medium
		GDP-IE7 ← CEP	0.922					
		GDP-IE8 ← CEP	0.749					
		GDP-IE9 ← CEP	0.764					
		GDP-IE10 ← CEP	0.922					
		GDP-IE11 ← CEP	0.700					
		GDP-IE12 ← CEP	0.906					
	CFP			0.916	0.941	0.8	0.566	Medium
		GDP-IE13 ← CFP	0.865					
		GDP-IE14 ← CFP	0.928					
		GDP-IE15 ← CFP	0.841					
		GDP-IE16 ← CFP	0.939					

SIL: standardized indicator loadings, CA: Cronbach’s alpha, CR: composite reliability, AVE: average variance extracted, LEP: level of explanatory power, CTR: corporate tangible resources, CIR: corporate intangible resources, MS: market environment, PS: public supervision, PIE: policy and institutional environment, GDB: green development behavior, GSCMP: green supply chain management practice, CPB: clean production behavior, GDP: green development performance, CSP: corporate social performance, CFP: corporate financial performance, CEP: corporate environmental performance, GDP-IE: green development behavior of industrial enterprises, GDP-IE: green development performance of industrial enterprises.

**Table 4:** Heterotrait–monotrait ratio (HTMT) values.

	CPB	CEP	CFP	CIR	CSP	CTR	GDB	GDP	GSCMP	ME	PIE	PS
CPB												
CEP	0.446											
CFP	0.473	0.441										
CIR	0.583	0.456	0.408									
CSP	0.517	0.574	0.588	0.648								
CTR	0.582	0.379	0.443	0.547	0.568							
GDB	0.851	0.594	0.558	0.702	0.685	0.639						
GDP	0.583	0.891	0.812	0.625	0.868	0.566	0.752					
GSCMP	0.575	0.636	0.521	0.674	0.729	0.54	0.888	0.778				
ME	0.457	0.403	0.398	0.472	0.563	0.476	0.566	0.56	0.56			
PIE	0.363	0.407	0.254	0.407	0.421	0.382	0.471	0.452	0.491	0.379		
PS	0.262	0.424	0.239	0.33	0.325	0.214	0.417	0.412	0.518	0.381	0.319	

**Table 5:** Path coefficients and confidence interval.

Hypothesis		H1	H2	H3	H4	H5	H6
**Path Relationships**		CTR → GDB	CIR → GDB	ME → GDB	PS → GDB	PIE → GDB	GDB → GDP
**Path coefficient (β)**		0.304	0.328	0.169	0.138	0.111	0.706
**Standard Error**		0.034	0.038	0.039	0.034	0.037	0.026
**Confidence Interval**	**5.0%**	0.248	0.267	0.106	0.078	0049	0.665
	**95.0%**	0.361	0.393	0.234	0.190	0.171	0.749
***f*^2^**	**Value**	0.148	0.176	0.046	0.038	0.023	0.996
	**Effect**	Moderate	Strong	Moderate	Moderate	Moderate	Strong
***t* Values**		9.009	8.595	4.370	4.071	3.009	27.557
***p* Values**		0.000	0.000	0.000	0.000	0.001	0.000
**Significance level**		***	***	***	***	**	***
**Result**		Supported	Supported	Supported	Supported	Supported	Supported

Note: path significance: ** *p* < 0.01, *** *p* < 0.001.

## Appendix A

**Table A1:** Questionnaire items on the internal factors (IFs) [22].

Dimensions	Codes	Items
Corporate tangible resources (CTR)	IFs1	Our enterprise is an organization with a high awareness and mission of green production.
	IFs2	Our enterprise has a leadership that values green production.
	IFs3	Our enterprise has a department or organization in charge of environmental work.
	IFs4	Our enterprise has a special budget for green production.
	IFs5	Our enterprise has a leadership that is committed to green production.
	IFs6	The sufficient capital level of our enterprise can support green production.
	IFs7	Our enterprise regularly trains employees in green production-related skills.
	IFs8	Our enterprise has sufficient talent reserves related to green production.
	IFs9	Our enterprise has production equipment that fully meets the needs of green production.
Corporate intangible resources (CIR)	IFs10	Our enterprise can easily design green ecological products.
	IFs11	Our corporate products have the ability to register the green logo.
	IFs12	Our enterprise has the ability to market green products.
	IFs13	In the field of green production, our enterprise has stocked related advanced technologies.

**Table A2:** Questionnaire items on the external factors (EFs) [22].

Dimensions	Codes	Items
Market environment (ME)	EFs1	The enforcement of green production-related regulations in the market is strict.
	EFs2	The implementation of green production-related systems in the market is strict.
	EFs3	Consumers trust green products.
	EFs4	Green production helps to enhance corporate image and brand value.
	EFs5	Consumers tend to buy green products.
	EFs6	In the market, enterprises are heavily regulated.
	EFs7	In the market, the green production of enterprises has been actively supported.
	EFs8	In the market, enterprises’ participation in the construction of ecological industrial parks has been actively supported.
	EFs9	The customer (enterprise) has high requirements for the environment of our enterprise.
	EFs10	Investors place high demands on the environmental protection of our enterprise.
	EFs11	In the market, green production-related regulations and systems are highly practical.
Public supervision (PS)	EFs12	Community residents are required to participate in the environmental impact approval process of surrounding enterprises.
	EFs13	The public and the community will make petition letters or complaints about environmental violations of surrounding enterprises.
	EFs14	Residents of the community require surrounding enterprises to build public environmental protection infrastructure.
	EFs15	Social environmental organizations are very concerned about corporate environmental violations.
	EFs16	News media will report on corporate environmental violations.
	EFs17	Peers are very concerned about the enterprise’s green production capabilities.
	EFs18	Consumers pay great attention to the environmental violations of enterprises.
	EFs19	Green products have passed strict certification.
Policy and institutional environment (PIE)	EFs20	The government has developed preferential land policies for enterprises adopting clean technologies.
	EFs21	The government has developed investment and financing policies for enterprises adopting clean technologies.
	EFs22	The government has developed fiscal and tax incentives for enterprises adopting clean technologies.
	EFs23	The government actively implements preferential policies for cleaner production of enterprises.

**Table A3:** Questionnaire items on the green development behavior of industrial enterprises (GDB-IE) [22].

Dimensions	Codes	Items
Clean production behavior (CPB)	GDB-IE1	The production process of our enterprise strictly adheres to the requirements of cleaner production.
	GDB-IE2	Our enterprise is selecting and improving pro-environmental processes or equipment.
	GDB-IE3	Our enterprise purchases environmentally friendly processes and equipment.
	GDB-IE4	Our enterprise considers the need for cleaner production when designing products.
	GDB-IE5	Our enterprise actively builds a cleaner production brand.
	GDB-IE6	Our enterprise has promoted the image of cleaner production.
	GDB-IE7	Our enterprise cascades use energy between enterprises.
	GDB-IE8	Our enterprise recycles water between enterprises.
	GDB-IE9	Our enterprise is actively looking for partners to jointly achieve the goals of energy conservation and emission reduction.
	GDB-IE10	Our enterprise actively recycles and disposes of waste products.
Green supply chain management practices (GSCMP)	GDB-IE11	Our enterprise conducts environmental and energy audits on the internal management of suppliers.
	GDB-IE12	Our enterprise requires suppliers to provide design specifications for the environmentally friendly requirements of the products they purchase.
	GDB-IE13	Our enterprise evaluates suppliers’ environmentally friendly practices.
	GDB-IE14	In the supply chain, our enterprise is very concerned about the green technologies of other enterprises.
	GDB-IE15	Our enterprise purchases new energy-saving and low-carbon materials and new energy.
	GDB-IE16	In the supply chain, our enterprise actively shares energy-saving and emission-reduction technologies among enterprises.
	GDB-IE17	Our company chooses suppliers that have passed third-party environmental management system certification (e.g., ISO 14001).
	GDB-IE18	In the supply chain, our enterprise actively communicates information about byproducts between enterprises.

**Table A4:** Questionnaire items on the green development performance of industrial enterprises (GDB-IE) [22].

Dimensions	Codes	Items
Corporate social performance (CSP)	GDP-IE1	Customers increasingly trust our products.
	GDP-IE2	Our enterprise’s image and brand value have been enhanced.
	GDP-IE3	Our enterprise has improved product quality.
	GDP-IE4	Stakeholders have a high opinion of our enterprise.
	GDP-IE5	Our enterprise has improved relationships with the people in our communities.
	GDP-IE6	Our enterprise reduces the possibility of environmental accidents.
Corporate financial performance (CFP)	GDP-IE7	Our enterprise has reduced material costs.
	GDP-IE8	Our enterprise has reduced operating costs.
	GDP-IE9	Our enterprise has reduced energy costs.
	GDP-IE10	Our enterprise has reduced the cost of environmental governance (e.g., emissions, penalties).
	GDP-IE11	Our enterprise has improved long-term financial performance.
	GDP-IE12	Our enterprise has reduced procurement costs through material recycling.
Corporate environmental performance (CEP)	GDP-IE13	Wastewater emissions from our enterprise have decreased.
	GDP-IE14	The emissions of exhaust gas produced by our enterprise have decreased.
	GDP-IE15	The amount of solid waste produced by our enterprise has decreased.
	GDP-IE16	Our enterprise has reduced the consumption of dangerous, toxic and harmful substances.
